# The Effect of Elastomer Content and Annealing on the Physical Properties of Upcycled Polyethylene Terephthalate-Maleated Styrene Ethylene Butylene Styrene Blends for Additive Manufacturing

**DOI:** 10.3390/ma17246272

**Published:** 2024-12-22

**Authors:** Diego Bermudez, Stephanie Moreno, David A. Roberson

**Affiliations:** 1 Polymer Extrusion Lab, The University of Texas at El Paso, El Paso, TX 79968, USA; dbermudez2@miners.utep.edu (D.B.); smoreno25@miners.utep.edu (S.M.); 2 Department of Metallurgical, Materials and Biomedical Engineering, The University of Texas at El Paso, El Paso, TX 79968, USA

**Keywords:** additive manufacturing, upcycling, shape memory polymers, polymer crystallinity, fused filament fabrication, 4D printing

## Abstract

In the work presented here, we explore the upcycling of polyethylene terephthalate (PET) that was derived from water bottles. The material was granulated and extruded into a filament compatible with fused filament fabrication (FFF) additive manufacturing platforms. Three iterations of PET combined with a thermoplastic elastomer, styrene ethylene butylene styrene with a maleic anhydride graft (SEBS-g-MA), were made with 5, 10, and 20% by mass elastomer content. The elastomer and specific mass percentages were chosen based on prior successes involving acrylonitrile butadiene styrene (ABS), in which the maleic anhydride graft enabled compatibility between different materials. The rheological properties of PET and the PET/SEBS blends were characterized by the melt flow index and dynamic mechanical analysis. The addition of SEBS-g-MA did not have a significant impact on mechanical properties, as determined by tensile and impact testing, where all test specimens were manufactured by FFF. Delamination of the tensile specimens convoluted the ability to discern differences in the mechanical properties, particularly % elongation. Annealing of the specimens enabled the observation of the effect of elastomer content on the mechanical properties, particularly in the case of impact testing, where the impact strength increased with the increase in SEBS content. However, annealing led to shrinkage of the specimens, detracting from the realized benefits of the thermal process. Scanning electron microscopy of spent tensile specimens revealed that, in the non-annealed condition, SEBS formed nodules that would detach from the PET matrix during the tensile test, indicating that a robust bond was not present. The addition of SEBS-g-MA did allow for shape memory property characterization, where deformation of tensile specimens occurred at room temperature. Specimens from the 20% by mass elastomer content sample group exhibited a shape fixation ratio on the order of 99% and a shape recovery ratio on the order of 80%. This work demonstrates a potential waste reduction strategy to tackle the problem of polymer waste by upcycling discarded plastic into a feedstock material for additive manufacturing with shape memory properties.

## 1. Introduction

Society’s widespread use of synthetic polymeric materials has steadily contributed to a global waste management problem that has become an increasingly alarming environmental crisis [1]. The United States (US) is currently faced with overwhelming amounts of waste production due to rampant use of single-use plastics coupled with imperfect municipal and nationwide management of solid waste material [1,2]. Moreover, the lack of efficient systems to properly dispose of or recycle plastic goods has resulted in unprecedented levels of waste being discarded in landfills and waterways, much of which is non-biodegradable waste [1,3].

Many of these dumping areas include natural environments that become long-term destinations for much of the discarded plastic waste. Evidence regarding the adverse effects that the gradual chemical and mechanical breakdown of plastics into the microscopic scale has on their surrounding environment is well documented; chemicals such as phthalates have been known to cause malignant effects in the endocrine systems of animals, affecting the reproductive trends of fish and wildlife. Gradual degradation of plastics will culminate in their ultimate assimilation into ecosystems. More expressly seen in waterways, the integration of microplastics and their accompanying derived chemicals have been known to cause harmful effects in maritime wildlife [4], and there are already records of their presence in the human body. Endocrine system attacks resulting from microplastics consumption does not stop with sea wildlife, as indirect consumption of microplastic-contaminated seafood is on the order of 11,000 particles per person, per year. What is more troublesome is that microplastics have been found in every ecosystem, meaning that their consumption is not limited to seafood eaters alone. Humans are now more broadly susceptible to consuming these plastics and being exposed to harmful chemicals like plasticizers which have been linked to fetal development issues in humans. More evidence of the presence of microplastics in humans include their detection as traces in the lung tissue of living humans, in the blood and tissue of human placentas, and in samples of fecal matter that have been linked to Inflammatory Bowel Disease [5,6,7,8,9,10,11,12,13].

The increase in the detection of microplastics in wildlife and in human tissue, coupled with ineffective waste disposal practices, elucidate an urgent need to seek alternative avenues that may extend the use of these materials beyond their primary service life cycle and away from end destinations that will result in environmental harm. Waste management is a challenge faced by several countries in which the amount of plastic waste generated globally was 6.9 billion tons and is expected to grow to 12 billion tons by 2050 [14]. In 2017 it was reported that, in the US, 12.2% of total waste solid generation (35,680 tons) was plastic waste; out of the 35,680 tons of plastic waste generated, only 3090 tons were recycled (8.6%), 5620 tons were combusted for energy release (15.8%), and 26,970 tons were landfilled (~75%) [15]. A different category focuses on waste management at the municipal level; of all municipal solid waste (MSW) landfilled in 2018, 27 million pounds of plastic were landfilled, constituting 18.5 percent of all landfilled MSW [15]. A material that is included in the category of plastics is polyethylene terephthalate (PET), which is commonly used to manufacture bottles for beverages. Although the overall amount of recycled plastic bottles is relatively small (with an 8.7 recycling rate in 2018), the amount of bottles specifically manufactured from PET recycled in the US was 29.1% in 2018 [14,15]; however, this is much lower than the recycling rate of PET bottles in other countries such as Norway, which recycled 97% of discarded PET bottles in the same year [14].

A previous study by our group explored the upcycling of PET bottles by creating a composite where cellulose fibers were extracted from denim, in an effort to divert material from two waste streams, plastic and discarded textiles, into usable feedstock for fused filament fabrication (FFF) 3D printers [2,5]. In this prior work, the impact resistance of FFF-made specimens was increased due to the addition of cellulose material. Other investigators have explored fiber-reinforcement of recycled polymeric materials, namely polypropylene (PP) [16,17], and it is conceivable that our PET/SEBS-g-MA blend could later be fiber-reinforced, but we have limited this study to only explore the effect of rubber content and heat treatment. In the context of upcycling PET water bottles, other works by Zander et al. [18] and Little et al. [19] demonstrated direct recycling of PET bottles into feedstock for extrusion-based additive manufacturing (AM) platforms without the incorporation of another material. The work presented here again demonstrates an upcycling approach for the creation of feedstock materials for the AM technology of FFF, but this time it incorporates a thermoplastic elastomer, maleic anhydride grafted styrene ethylene butylene styrene (SEBS-g-MA) in the creation of a polymer blend.

The work presented here is part of a larger body of work by Bermudez found in [20]. We chose to incorporate SEBS-g-MA (hereinafter referred to more simply as SEBS) because it has been shown to provide benefits in terms of a toughening mechanism when combined with PET [21]. Moreover, our group has demonstrated the compounding of SEBS (both maleated and non-maleated) with a wide variety of rigid thermoplastics, including acrylonitrile butadiene styrene (ABS) [12,22,23,24,25,26,27,28], polylactic acid (PLA) [13,29,30], and polycarbonate (PC) [31]. In particular, an early work conducted by Siqueiros et al. [25] demonstrated a blend of ABS and SEBS-g-MA to be suitable for the fabrication of impact-dampening structures. All our previous efforts dealt with the creation of FFF feedstock. The overarching goal of this work is to explore strategies for upcycling material derived from PET water bottles through the addition of thermoplastic elastomer and/or heat treatment processes.

**Figure 1:**
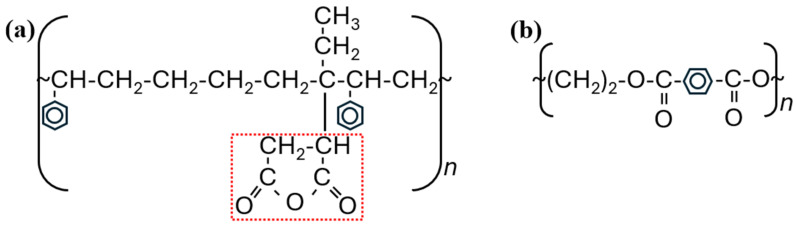
Representation of the chemical structure of (**a**) SEBS-g-MA and (**b**) PET.

For this work, we chose to use a variant of SEBS with a maleic anhydride graft to accommodate structural differences between SEBS and PET. We note here that PET is a long-chain semicrystalline polyester [32] and SEBS-g-MA is a copolymer triblock that has an additional graft functional group that improves its compatibility with dissimilar materials due to the reactivity of the group [33]. Representative depictions of both PET and SEBS-g-MA are presented in Figure 1, where the maleic anhydride graft attached to SEBS is highlighted by a red dashed box (Fig 1a).

## 2. Materials and Methods

For this study, PET bottles obtained from Cordova Distributing Company (Cordova Distributing Company, LLC, El Paso, TX, USA) were used. The material was received in the form of empty bottles with no caps. We chose unused plastic bottles as our base material due to the potential health and safety risks associated with working with used food and beverage containers. The SEBS used in this study (Grade FG1901-GT, Kraton, Houston, TX, USA) was obtained in pellet form. PET processing began with shredding the bottles using a Filabot Reclaimer granulator (Filabot, Barre, VT, USA) equipped with a 5 mm screen size sifter. The larger particles were segregated from the smaller, sieved granules to leave a reliably homogenous particle size distribution with the intent of facilitating the extrusion process. Considering that PET, like other polyesters such as PLA, can be subject to a degradation in its mechanical properties due to hygroscopicity [34,35], drying cycles were run prior to the melt processing of PET and PET-SEBS to reduce the detrimental effect of moisture absorption. The drying schedule consisted of holding the constituents to a 4 h, 130 °C cycle inside a VWR Signature horizontal air flow oven (Model 1370FM, VWR International, West Chester, PA, USA) and was performed for granulated materials and on the extruded filament. The PET granulate was extruded into 3D-printable monofilaments using a Filabot EX2 single-screw extruder. During the extrusion process, the filaments were air-cooled using a Filabot Airpath. Extruding temperatures ranged from 260 °C to 265 °C with a screw speed set at the maximum available speed on this model (~35 rpm), as shown in Table 1. To achieve a relatively constant diameter, a belt puller (Collin Teachline, Collin Lab and Pilot Solutions, Norcross GA, USA) was used to draw and wind the filament.

Melt flow indexing was performed using a Tinius Olsen MP1200 melt flow indexer (Tinius Olsen, Horsham, PA, USA) to characterize the rheological properties of PET, and the blends examined in this work (where % SEBS is by mass): PET/5% SEBS, PET/10% SEBS, and PET/20% SEBS. The melt flow index (MFI) was measured in accordance with ASTM D1238 Procedure A [36], using approximately 6–8 g of material for each run; all material types were tested three times (n = 3).

Fourier transmission infrared spectroscopy in attenuated total reflectance mode (FTIR-ATR) was used to characterize the chemical groups belonging to each polymer constituent, as well as for each polymeric blend of PET and SEBS. A Nicolete™ iS™ 5 FTIR equipped with an iD7 attenuated total reflectance (ATR) diamond (Thermo Fisher Scientific, Waltham, MA, USA) was used to collect the materials’ spectra at a collection duration of 30 s.

**Figure 2:**
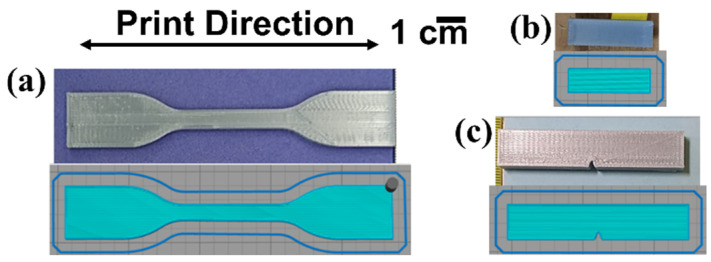
Examples of additively manufactured test specimens for (**a**) tensile testing, (**b**) DMA testing and (**c**) Izod impact testing.

All test specimens were additively manufactured using a Lulzbot TAZ 5 SE FFF (Fargo Additive Manufacturing Equipment, Fargo, ND, USA) with a 0.5 mm nozzle at a temperature of 265 °C and bed temperature of 70 °C. Simplify 3D V4 (Simplify 3D, Cincinnati, OH, USA) software was used to establish print parameters and prepare the g-code to be used by the FFF 3D printer. All print rasters were oriented in a longitudinal direction (parallel with the length of the given test specimen) as shown in Figure 2. The printing parameters are listed in Table 2. The dimensions of the tensile test specimens were based on the ASTM D638 standard for the tensile testing of plastics, specifically the Type IV geometry [37], while the dimensions for impact test specimens were based on those specified in the ASTM D256 standard for the Izod impact testing of plastics [38] and, finally, the dimensions for DMA specimens were 3 mm × 9 mm × 30 mm, in line with what is recommended in the ASTM D5418 standard, which indicates that a rectangular specimen of any dimension can be used as long as the dimensions are reported [39].

Tensile testing was performed using an MTS Criterion C.44 (MTS, Eden Prairie, MN, USA) tensile tester with an Advantage Model AHX 800 high elongation extensometer, at a rate of 5 mm/min as specified by standard for semirigid classification Type IV specimens [37]. Samples were pulled until failure and the exposed fracture surfaces were protected for subsequent fractography analysis in the scanning electron microscope (SEM). 

Analysis of the fracture surfaces of tensile test specimens was carried out with a Hitachi SU-3500 (Hitachi High-Tech America, Inc., Schaumburg, IL, USA) variable-pressure SEM equipped with ultra-variable pressure (UVD) and backscatter electron (BSE) detectors. To mitigate the effects of electron charging, the instrument was operated at a pressure ranging from 30 to 110 Pa.

In this study, we also carried out annealing experiments where the annealing schedule entailed holding the specimens at a temperature of 130 °C for four hours and then letting the specimens cool down in the same horizontal air flow oven used for drying. The effect of annealing was partly characterized by way of X-ray diffraction (XRD) using a Bruker D8 Discover XRD (Bruker, Madison, WI, USA) with a Cu K_α_ (λ  = 1.54 Å) X-ray source.

**Figure 3:**
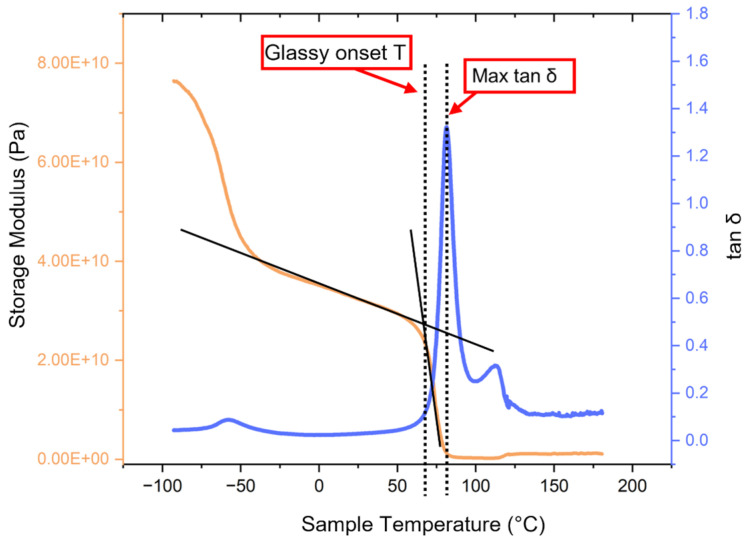
Determination of the glassy onset temperature and the temperature at which the max tan δ occurs.

Dynamic mechanical analysis (DMA) consisted of running a dual cantilever mode temperature sweep from −80 °C to 180 °C at a heating rate of 2 °C/min and frequency of 1 Hz, on a Perkin Elmer DMA 8000 (Perkin Elmer, Waltham, MA, USA). The glassy onset of the material was determined based on the procedure described in the ASTM E1640 standard [40]. The DMA test was also used to determine the temperature at which the max tan δ was reached, as this is pertinent to shape memory property testing. The machine parameters for the DMA test are listed in Table 3 and an example of the calculation of T_g_ based on the glassy onset temperature and the determination of the temperature at which max tan δ occurs can be seen in Figure 3.

Prior to shape memory analysis, preliminary impact and tensile testing was performed using samples printed using both neat PET and the range of PET-SEBS polymeric blends. Shape memory testing was performed on specimens made from the blend that could sustain the greatest % elongation without failure of the specimen. The typical methodology for characterizing the shape memory properties, namely the shape recovery ratio (*R_r_*) and shape fixation ratio (*R_f_*) found in the literature for heat-activated shape memory polymers [41,42,43,44] consists of the following steps: (1) the specimen is heated to a temperature marginally below the T_g_; (2) the specimen is deformed into a temporary distorted shape and then allowed to cool; and (3) the sample is heated to a temperature above its T_g_ to recover its original shape. We have shown in a previous work that the glassy onset temperature, as determined by DMA, is suitable for a deformation temperature and the temperature at which the max tan δ occurs is suitable for a recovery temperature [29].

Considering that the service temperature for most polymeric materials is room temperature, the procedure employed in this work deviated from the typical characterization technique. In this work, the deformation did not occur at an elevated temperature, but rather at room temperature. The specimen type used to characterize the shape memory properties was the ASTM D638 Type IV Tensile specimen. For shape memory property analysis, several test coupons were printed, then deformed at room temperature on the tensile tester, and finally recovered at a temperature above the T_g_. The following equations are generally used to calculate the shape memory properties as documented elsewhere in literature [12,27,41,42]. The *R_f_* value is calculated by the following equation:

(1) $$R_{f}(\%)=\frac{\varepsilon_{u}}{\varepsilon_{m}}\times 100\%$$

where $\varepsilon_{m}$ is the maximum % elongation and $\varepsilon_{u}$ is the elongation that remains after the load is removed from the specimen, quantifying the material’s ability to sustain and maintain shape deformation. The *R_r_* value is calculated by the following equation:

(2) $$R_{r}(\%)=\frac{\varepsilon_{m}-\varepsilon_{p}}{\varepsilon_{m}}\times 100\%$$

where $\varepsilon_{p}$ is the elongation of the specimen after the recovery process, which is driven, in this case, by applied heat. Finally, shape memory index (*SMI*) is a value used to quantify the overall shape memory capability of a polymer and can be calculated as follows:

(3) $$SMI\;\left(\%\right)=(R_{r}\times R_{f})\times 100\%$$

The typical methodology for calculating the shape memory parameters entails pulling the specimen to 100% elongation ($\varepsilon_{m}$ = 100%). However, we found in preliminary tensile tests that our specimens could not sustain this level of strain without failure, so we modified our procedure to elongate the specimens to only 20% maximum elongation for shape memory testing. Additionally, an extensometer complication led to the inability to accurately record strain data, so we had to rely on the data provided by the tensile tester’s crosshead displacement to calculate strain. The utilization of displacement data required us to develop modified equations to quantify the shape memory properties. The equation used in this work to calculate the shape fixation ratio using displacement (*R_f_*_2_) was

(4) $$R_{f2}(\%)=\frac{l_{u}}{l_{m}}\times 100\%$$

where $l_{u}$ denotes the total length of the tensile sample after unloading it from the maximum elongation condition, $l_{m}$. To obtain a recovery ratio value, displacement was also used. Additionally, the factors of strain used in the equation were modified to replace the difference in maximum strain and strain after recovery, to the difference in lengths of the unloaded specimen, $l_{u}$ minus the length of the tensile specimen after its temperature recovery, $l_{p}$. The denominator portion of the new recovery ratio consisted of the difference between the length of the unloaded specimen and the original length, $l_{0}$. These modifications led to a new equation describing the recovery ratio using displacement (*R_r_*_2_):

(5) $$R_{r2}=\frac{l_{u}-l_{p}}{l_{u}-l_{o}}\times 100\%$$

Finally, a new equation was then used to calculate the shape memory index (*SMI*_2_):

(6) $${SMI}_{2}\left(\%\right)=\left(R_{r2}\times R_{f2}\;\right)\times 100\%$$

All specimens were subjected to a dwell time of 1 min to mitigate the shrinkage/recoil effects of elastic deformation and therefore obtain a consistent *R_f_*_2_; the samples were then released from the tensile machine grips and their displacement was measured with a caliper. The dwell time effectively leads to stress relaxation of the polymer, which elongates the chains [45]. The values for *R_r_*_2_ were obtained by subjecting the specimens of each PET-SEBS blend to a recovery cycle in the horizontal air flow oven at the temperature at which the max tan δ was observed for a period of 5 min.

## 3. Results and Discussion

### 3.1. Melt Flow Index

**Figure 4:**
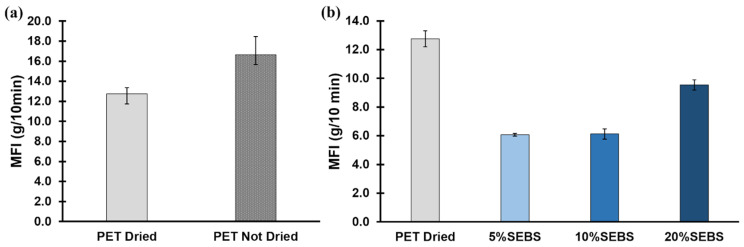
(**a**) MFI results comparing dried PET and PET that was not subjected to a drying cycle. (**b**) MFI test results comparing dried pet with the iterations of PET-SEBS studied in this work where all specimens were dried prior to testing.

Baseline results for the melt flow rate of dried and undried PET are graphically represented in Figure 4. Dried PET granules had a lower flow rate as opposed to the non-dry PET, which is demonstrative of hygroscopicity, as mentioned previously. In this case, the moisture effectively lowers the molecular weight of the PET due to chain scission, decreasing the viscosity and leading to more material exiting the MFI instrument during the test [34]. The measurements were made on specimens obtained from pelletized filaments, but not subjected to a drying cycle prior to MFI measurements. Thereinafter, MFI measurements were made on specimens that were dried. Figure 4b represents the MFI measurements for dried PET and dried PET/SEBS blends, where it is notable that the MFI for the of 5% (by weight) SEBS blend is statistically significantly lower than that observed for neat PET. The MFI values increased as the SEBS content increased, creating an upward trend in MFI. This initial drop of values indicates interference of molecular chain movement of the PET polymer chains by the SEBS component, effectively lowering the viscosity. There is little distinction between the MFI values of the 5% SEBS blend compared to the 10% SEBS blend with a difference in averages of only 0.05 g/10 min within the error. A significant change was observed in MFI values when comparing the 10% SEBS blend to the 20% SEBS blend, with the value of the latter having registered an average of 9.54 ± 0.35 g/10 min compared to the former, which had a value of 6.12 ± 0.36 g/10 min. The increase in MFI observed for the 20% (by weight) concentration of SEBS may indicate that the lower overall viscosity of SEBS compared to PET is driving the rheology of the material at this SEBS concentration, as the published MFI value for this grade of SEBS is 22 g/10 min [46]. The more profound effect on MFI values observed for the 20% by mass concentrations of SEBS may indicate a threshold in terms of elastomer concentration where the rheological properties of the second component begin to dominate the overall behavior of the blend.

### 3.2. Dynamic Mechanical Analysis

**Figure 5:**
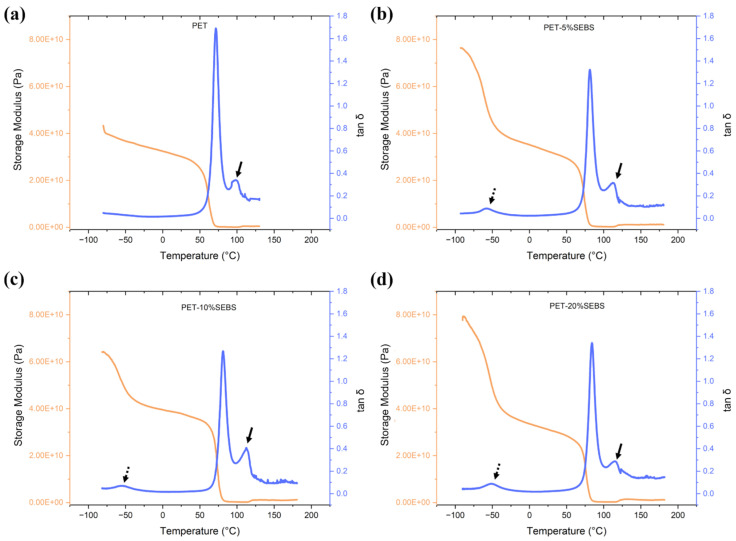
DMA curves for (**a**) neat PET, (**b**) PET/5% SEBS, (**c**) PET/10% SEBS, and (**d**) PET/20% SEBS.

As can be seen in the DMA plots in Figure 5, the PET component is a key driver of the rheological behavior of the blends, as a double peak manifests on the tan δ curve in each case, including the neat PET, with the second peak (indicated by the solid black arrows) indicative of the crystallization temperature of PET with a maxima in the range of 120 °C [47]. Also notable on the tan δ curves is a peak at ~−50 to −40 °C (highlighted by the dashed black arrows) which is due to the SEBS content, which has a T_g_ of −40 °C [29].

Table 4 contains the DMA data and allows for comparison of the rheological behavior of the blends. All materials tested exhibited a similar glassy onset temperature of ~67 °C. The glassy onset temperatures were 65.9 ± 2.2 °C, 69.1 ± 1.8 °C, 67.7 ± 1.1 °C, and 68.7 ± 2.3 °C for PET, PET/5% SEBS, PET/10% SEBS, and PET/20% SEBS, respectively. The highest value of storage modulus at glassy onset (measured in GPa), was exhibited by the PET specimens with an average value of 31.2 ± 7.0 GPa. The PET/SEBS blends displayed a decrease in average storage modulus, relative to PET, but within error of one another and PET, except for the 20% by weight SEBS blend. The storage modulus values for PET/5% SEBS, PET/10% SEBS, and PET/20% SEBS were 21.20 ± 7.2 GPa, 26.5 ± 8.4 GPa, and 22.2 ± 0.2 GPa, respectively.

In the case of tan δ values, the addition of SEBS lessens the dampening ability compared to PET. For neat PET, the tan δ was 1.6 ± 0.2. The values for PET/5% SEBS, PET/10% SEBS, and PET/20% SEBS were 1.3 ± 0.1, 1.3 ± 0.1, and 1.2 ± 0.1. These values were within a similar error range as the values for PET. Additionally, the temperatures at which max tan δ occurred were also similar between PET and the PET/SEBS blends on the order of 82 °C. As was mentioned previously, we have shown the temperature at which max tan δ occurs to be suitable as a temperature for the shape recovery process during shape memory property testing [29].

### 3.3. X-Ray Diffraction

**Figure 6:**
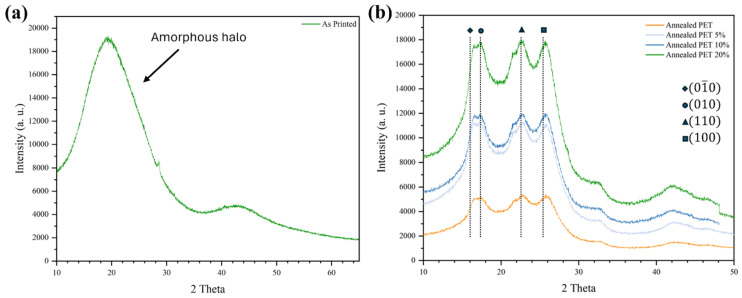
(**a**) XRD spectra for as-printed PET and (**b**) XRD spectra for annealed PET and the PET/SEBS blends studied here.

One aspect we wished to explore in this work was the effect of annealing on the physical properties of the upcycled PET/SEBS blend. The motivation behind this was to elucidate whether crystalline domains would lead to an improvement in physical properties. Here, we used XRD to determine the effect of annealing. As-printed PET specimens exhibited an amorphous halo (Figure 6a) indicating the lack of crystalline domains. Annealed PET and annealed specimens of the PET/SEBS blends exhibited broad peaks near the known reflections for PET (Figure 6b). The peaks present at or near 16°, 17.5°, 22.5°, and 25.5° correspond to the planes (0$\bar{1}$0) (010), (110) and (100) [48]. It has been demonstrated that PET can achieve crystallinity by the formation of spherulitic domains with a triclinic crystal structure [49].

### 3.4. FTIR-ATR

**Figure 7:**
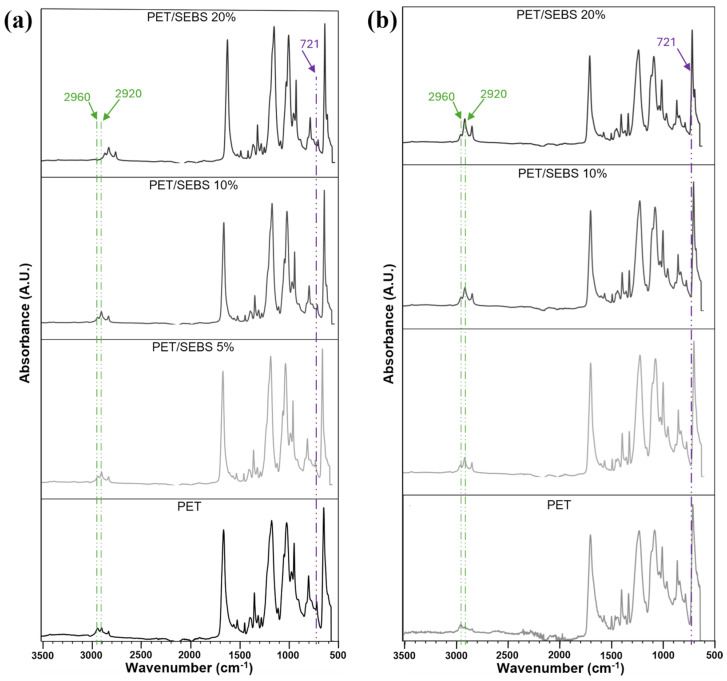
ATR spectra of PET and PET/SEBS blends in the (**a**) as-printed and (**b**) annealed conditions.

The addition of SEBS to PET can be seen in the FTIR-ATR spectra (Figure 7). A rightward shift is seen at 2960 cm^−1^ and 2920 cm^−1^ when SEBS is added to PET. This region corresponds with the stretching of the C-H bond in PET The wavenumber 2960 cm^−1^ corresponds to a C-H asymmetry stretching of the ethylene–butylene (EB) block of SEBS, while 2920 cm^−1^ corresponds to C-H asymmetry stretching in the EB block and C-H asymmetry stretching of the fundamental chain in the styrene (S) end blocks of SEBS [50], confirming the presence and influence of SEBS in the polymeric blend. Moreover, a nascent peak at 700 cm^−1^ becomes progressively more pronounced as SEBS content increases, and another rightward shift is seen at 721 cm^−1^ in the spectra corresponding with the blends. These peaks are associated with the C-H bending vibration of the side benzene rings of the S block, and to a C-H rocking of the -CH_2_ component of the EB block, respectively [51,52]. The characteristic peaks of SEBS appear at relevant wavenumbers. In general, the as-printed PET-SEBS spectra differed from the PET spectrum in that there is a slight shift to the right for many of the larger peaks, the absorbance was also generally increased upon the addition of SEBS, and finally new peaks characteristic of SEBS appeared in the spectra, with special emphasis on weak and mid-level peaks. Annealing the specimens caused the shifts observed at 2960 and 2920 cm^−1^ to disappear (Figure 7b). Moreover, the peak associated with the S block at 700 cm^−1^ disappeared. The difference in spectra between the annealed and non-annealed states indicates a chemical difference caused by the annealing process.

### 3.5. Mechanical Testing

**Figure 8:**
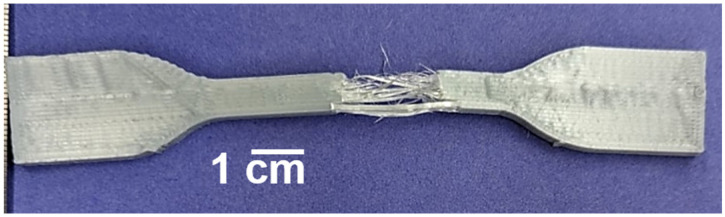
Example of delamination of a tensile test specimen.

Tensile testing revealed certain trends associated with the percentage of SEBS used in each of the batches. Overall, on average, the ultimate tensile strength (UTS) showed a decrease in strength as the SEBS content increased. Neat PET showed the highest UTS values of 51.0 ± 6.6 MPa; subsequently, the average of each category saw a progressive decrease, culminating with the 20% SEBS blend, which had the lowest average UTS (35.45 ± 6.2 MPa) but also showing the highest variability of results for as-printed specimens expressed in this blend’s standard deviation. Two sample pools showed this wide range of data: Neat PET and 20% SEBS, while 10% SEBS showed the least variability in this mechanical property. The decrease in UTS as the SEBS content increased was most likely due to a lack of miscibility between PET and the elastomer. The Hildebrand solubility parameter (δ) for SEBS is on the order of 17 MPa^1/2^, [53] while the δ for PET ranges from 20.5 to 23.0 MPa^1/2^ [54], meaning that any blend composed of these two materials would be immiscible. In terms of strain, the % elongation showed a large range of results for the blend iterations of 5% SEBS (17.86 ± 13.1%) and 20% SEBS (16.90 ± 10.6%), while 10% SEBS (8.35 ± 0.3%) demonstrated low elongation values and more consistent results. Finally, 10% SEBS showed the lowest strain out of all blends, including neat PET, and overall, low elongation values were obtained for all blends and no sample surpassed a strain value of 35%. The high variability seen across the different blends and in the values for the categories of tensile properties can be attributed to printing defects affecting the mode of failure during tensile testing, namely, delamination, an example of which is seen in Figure 8. During tensile testing, the instrument halts the test once a break is registered; in the cases of 5% SEBS and 20% SEBS tensile samples, the variable elongation registered was attributed to delamination, during which the software interpreted premature failures upon the onset of delamination, thus lowering the lower end of the % elongation range dataset.

**Figure 9:**
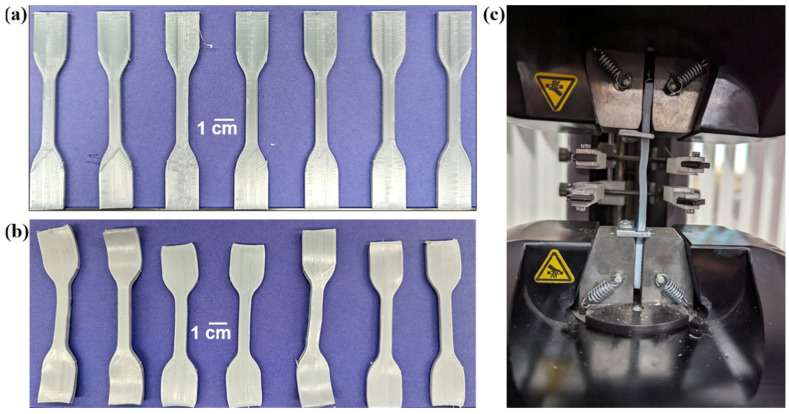
Examples of FFF made tensile specimens in (**a**) the as-printed condition and (**b**) after annealing; (**c**) annealed specimen in the tensile tester.

We must point out here that annealing of the specimens led to specimen shrinkage and warpage, which would most likely negate any improvement in physical properties for a part intended for service. An example of the shrinkage can be seen in Figure 8, where a batch of specimens is shown in the as-printed and annealed conditions (Figure 9a and 9b, respectively). The distortion of the specimens made tensile testing of the specimens challenging (Figure 9c); however, we have done our best to generate comparative data to understand the effects of annealing on the mechanical properties of PET and PET/SEBS blends.

**Figure 10:**
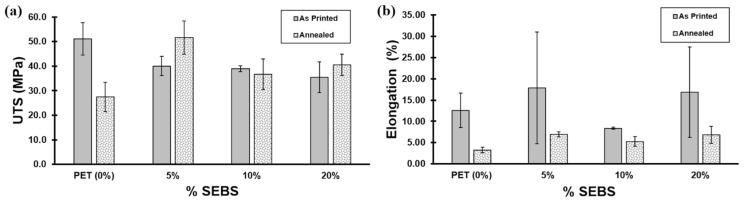
Tensile test results: (**a**) UTS values for as-printed and annealed specimens and (**b**) % elongation values for as-printed and annealed specimens.

Annealing pure PET specimens led to nearly a 50% reduction in UTS values, where the UTS for the annealed specimens was 27.4 ± 6.0 MPa compared to 51.0 ± 6.6 MPa for specimens that were not annealed. In contrast, the PET/5% SEBS blend exhibited a statistically significant increase in UTS values, where annealed specimens yielded UTS values of 51.58 ± 6.8 MPa compared to 40.06 ± 3.9 MPa for non-annealed specimens, a difference slightly greater than the error (in terms of standard deviation). The effect of annealing on the PET/10% SEBS and PET/20% SEBS blends yielded results, in terms of UTS, that were not greater than the error, effectively having a negligible effect on the strength of the blend. In terms of % elongation, annealing led to an overall reduction in ductility of the specimen in the case of pure PET and the three PET/SEBS blends evaluated here. The results of tensile testing are tabularized in Table 5 and graphically represented in Figure 10.

### 3.6. Izod Impact Testing

**Figure 11:**
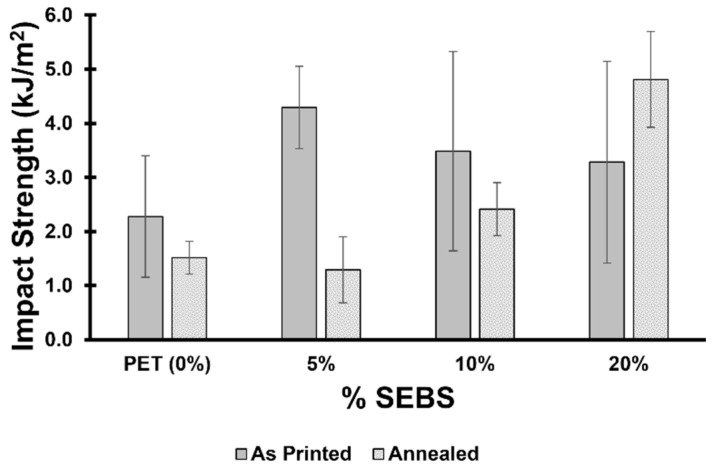
Impact test results in terms of impact strength for the blends studied here in the as-printed and annealed conditions.

It would be expected that adding elastomeric material to a rigid polymer, in this case, PET, would lead to rubber toughening of the material and that this effect would increase with an increase in elastomer content. However, in terms of impact strength, a significant increase in this property (as compared to PET) was only realized for the PET/5% SEBS blend. While the average impact strength of 10% and 20% by weight SEBS (3.5 ± 1.8 kJ/m^2^ and 3.3 ± 1.9 kJ/m^2^) was greater than the average impact strength of PET (2.3 ± 1.1 kJ/m^2^), the differences were within the standard deviation of the sample pools. Annealing led to a behavior that was more in line with expectations, where impact strength trended with an increase in SEBS content; however, the annealed sample pool of PET/5% SEBS specimens yielded a lower average impact strength than PET with a value of 0.8 ± 0.6 kJ/m^2^ compared to 1.1 ± 0.3 kJ/m^2^—within error of one another. One reason for the atypical behavior with respect to rubber toughening may once again be due to the differences in solubility parameter between the two materials as was the case with the UTS results. The annealing process possibly leads to a greater coalescence of the two materials, as demonstrated by the more typical behavior in terms of an increase in impact strength resulting from an increase in elastomer content and a decrease in the variability in data within the sample pools. The impact test data are graphically represented in Figure 11.

### 3.7. Fractography of Tensile Specimens

**Figure 12:**
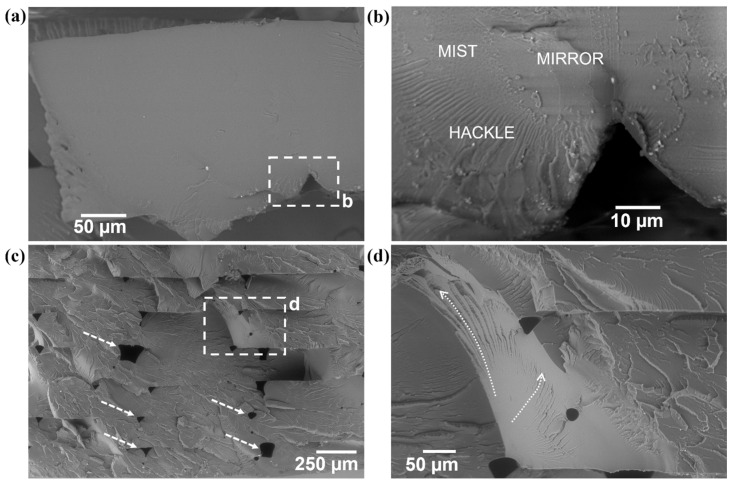
Fracture surfaces of (**a**) as-printed PET tensile specimens where (**b**) is the higher-magnification area blocked out by the dashed box in (**a**), and (**c**) fracture surface of an annealed PET specimen where (**d**) is the area indicated by the dashed box in (**c**).

Microanalysis via SEM of the fracture surfaces of spent tensile specimens offered insight into the effect of adding SEBS as well as the effect of annealing. Beginning with the PET specimens (Figure 12), the fracture surface of a representative baseline specimen exhibits a fracture surface morphology that is consistent with a brittle material. Though delamination of the specimens did occur, analysis of the ends of the delaminated components was possible. The fracture surface is largely planar, and the region next to a print defect (indicated by the dashed box) possesses fracture surfaces features typical of a brittle fracture, namely a mirror, mist, and hackle zone, as seen in the higher-magnification image (Figure 12b). A stress field manifested at the cusp between two print beads and the local propagation of a crack occurred in this region [55]. Annealing the PET further embrittled the material. The fracture surface (Figure 12c) is dominated by craze cracking. Print defects (highlighted by white dashed arrows) seem not to have affected the crack propagation. A cleavage step (indicated by the dashed white box in Figure 12c and at a higher magnification in Figure 12d) is present, indicating that the final rupture occurred on different planes and in different directions [56].

**Figure 13:**
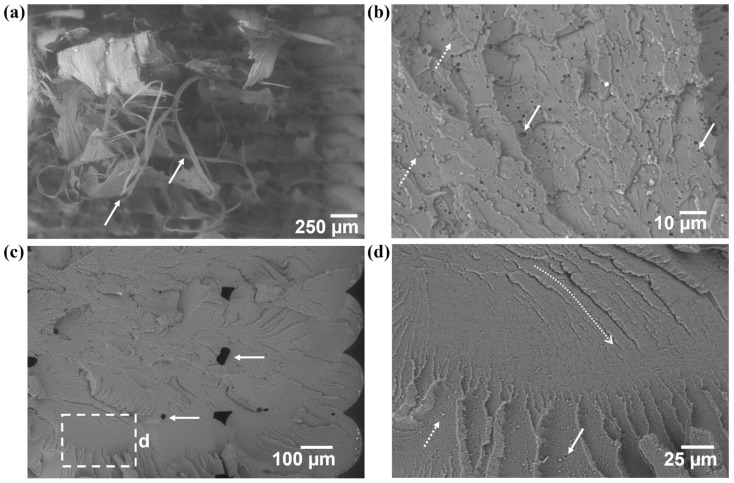
(**a**,**b**) Fracture surfaces of as-printed PET/5% SEBS tensile specimens and (**c**) fracture surface of an annealed PET/5% SEBS specimen where (**d**) is the area indicated by the dashed box in (**c**).

The addition of SEBS at a 5% mass content led to the manifestation of a mixed-mode fracture surface. Fibrils are present (Figure 13a); these are generally associated with ductile mode failure [12]. In a different region of the same specimen (Figure 13b), morphology more representative of craze cracking is also observed. The brittle-like morphology may be due to a localized reduction in the cross-sectional area leading to a rapid rupture. On this brittle surface, nodules that are most likely composed of SEBS (highlighted by dashed white arrows) and cusps (highlighted by solid white arrows) that are most likely tear-out sites for SEBS particles are also visible. The fracture surface of an annealed specimen of PET/5% SEBS (Figure 13d) exhibits a brittle-like morphology with features consistent with craze cracking. Print defects (highlighted by solid white arrows) are visible. Another mist and hackle zone (outlined by the white dashed box and seen in higher magnification in Figure 13d) is also present with cleavage steps that are parallel with the local crack propagation direction.

**Figure 14:**
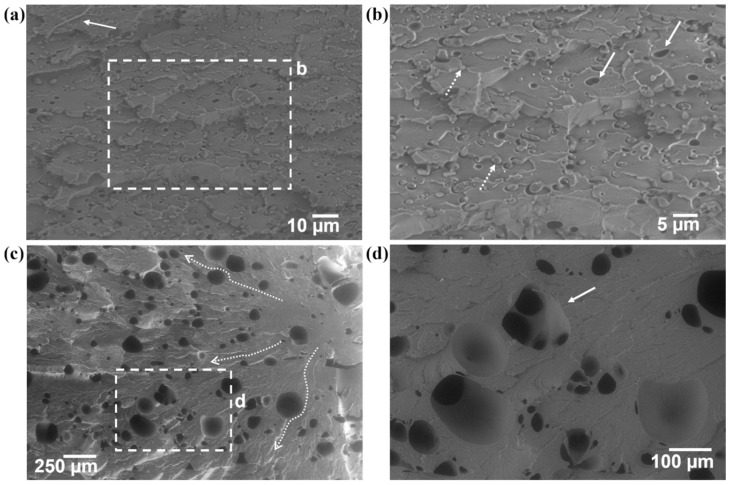
Fracture surfaces of (**a**) as-printed PET/10% SEBS tensile specimens where (**b**) is the higher-magnification area blocked out by the dashed box in (**a**), and (**c**) fracture surface of an annealed PET/10% SEBS specimen where (**d**) is the area indicated by the dashed box in (**c**).

The fracture surface of the PET/SEBS blend with 10% mass SEBS (Figure 14a) exhibited a similar fracture surface to the 5% mass blend where the fracture surface exhibited features indicative of craze cracking. A fibril (pointed out by the solid arrow in Figure 14a) was also present, indicating a mixed-mode ductile/brittle failure mechanism. The higher-magnification image (indicated by the dashed box in Figure 14a and seen in Figure 14b) again shows both SEBS nodules as well as voids where SEBS tore out during the tensile test, indicated by dashed and solid white arrows, respectively. Annealing the material led to the manifestation of craze cracking with less plastic deformation compared to the non-annealed specimens. The UVD image (Figure 14c) highlights the craze crack features due to the signal detected being analogous to that created by secondary electrons. The local crack propagation direction is indicated by the dashed arrows. There are voids present on the fracture surface of this specimen, but they are not caused by the tearing out of SEBS nodules. The higher-magnification image (Figure 14b) clearly shows secondary voiding (indicated by the solid white arrow), indicating that the voids were caused by a gas [24]. The gas could be the result of moisture or an element that volatized. We are unsure as to the actual cause of the voids, but it is possible that they are a result of an increase in elastomer content in combination with the annealing process, which we believe could lead to the emission of volatile organic compounds from the SEBS.

**Figure 15:**
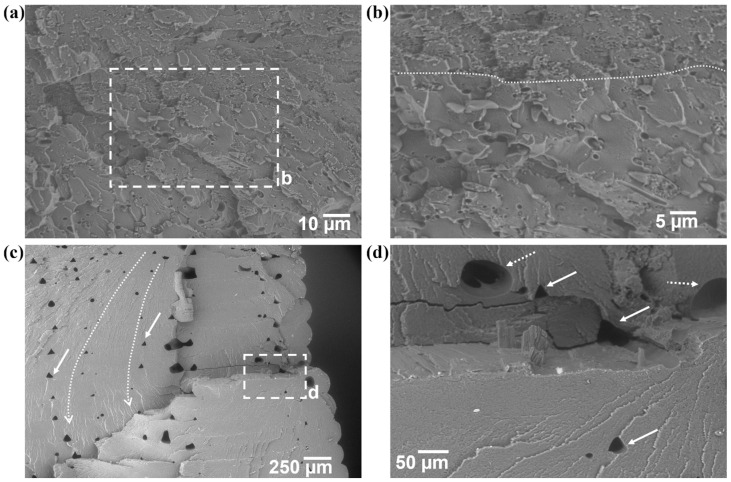
Fracture surfaces of (**a**) as-printed PET/20% SEBS tensile specimens where (**b**) is the higher-magnification area blocked out by the dashed box in (**a**), and (**c**) fracture surface of an annealed PET/20% SEBS specimen where (**d**) is the area indicated by the dashed box in (**c**).

A similar morphology was observed on the representative specimen from the 20% mass SEBS blend as was observed for the other two blends (Figure 15a). Again, a craze-cracking dominated fracture surface was present, and nodules of SEBS and tear-outs of SEBS are observed. There appeared to be a difference in the size distribution of the SEBS nodules, and there is a discernible distinction (indicated by the dashed line in Figure 15b) between one distribution and another. Annealing again embrittled the material, and the morphology was consistent with that of a brittle material despite the indication of SEBS. The crack propagation of a local crack is indicated by the dashed curved arrow and printing defects (indicated by solid white arrows) were also observed. A cleavage step (highlighted by a dashed white box and seen at a higher magnification in Figure 15d) was also observed. Voids were also present on the fracture surface and a distinction can be made between gas-induced and print-defect-related voids. The gas voids (indicated by the dashed white arrows in Figure 15d) are more rounded compared to the print defects (indicated by the solid white arrows) are more triangular. Additionally, secondary cracking appears to be initiated by the presence of the print-induced voids. The observation of gas-caused voids on the 20% by weight blend reinforces the notion that the SEBS content in combination with the annealing process may be the cause of this defect type.

### 3.8. Shape Memory Property Characterization

**Figure 16:**
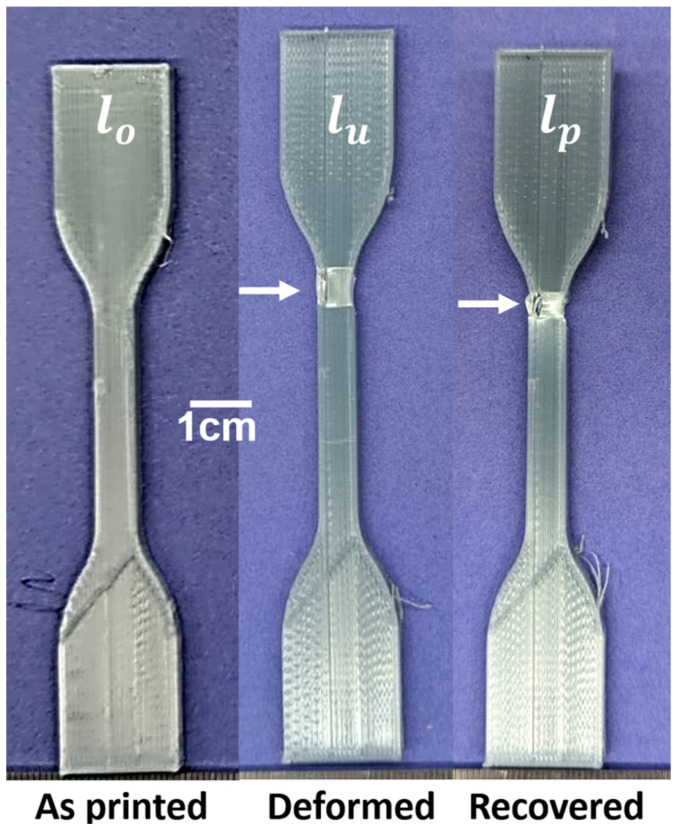
Example of the shape memory testing of a specimen composed of the PET/20% SEBS blend.

**Figure 17:**
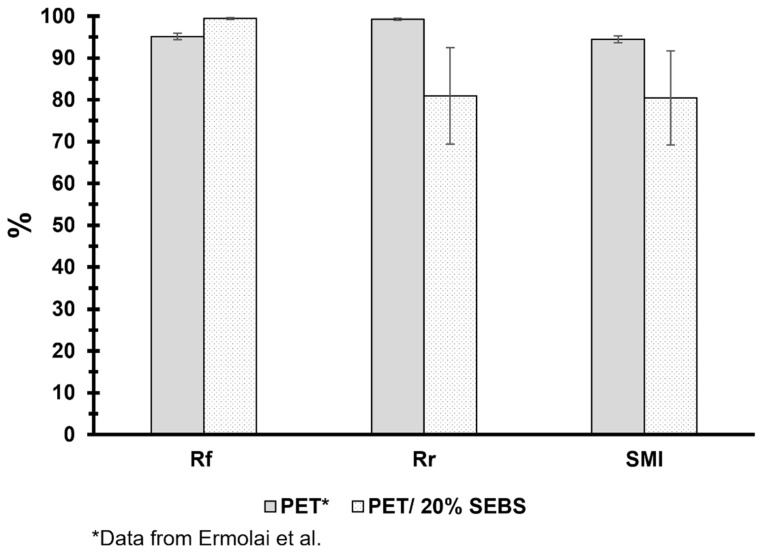
Calculated shape memory parameters for the PET/20% SEBS blend compared with data published by Ermolai et al. [57].

Tests to evaluate the shape memory properties were performed on a total of six specimens composed of PET/20% SEBS. Considering that SEBS is a soft elastomer, the mechanism of shape memory function would be considered a dual-component mechanism, where the PET is the hard component, which is responsible for the specimen returning to a permanent shape, and SEBS is the soft component, which is responsible for the holding of the temporary shape [29,41,45]. An example of the different steps at which the specimens were measured is shown in Figure 16. As can be seen in the figure, the deformation process induced a level of damage to the specimen that did not recover during the deformation process (indicated by white arrows). However, displacement-based calculations, as described previously, indicate that the *R_f_* of our specimens was 99.4 ± 0.2%. Work conducted by Ermolai et al. [57] which explored the shape memory properties of PET, where FFF-made specimens were deformed and recovered at an elevated temperature (80 °C), serves as a comparison to the work performed here. We remind the reader that our work differs from this cited work in that our shape memory process involved room temperature deformation to only 20% elongation (compared to 100% for the cited work) followed by an oven recovery step at a temperature of ~82 °C. Comparing our results to this cited work shows that the addition of SEBS increases the material’s ability to maintain a temporary shape (Figure 17). This observation is notable considering that the comparative work involving PET entailed the deformation of specimens at an elevated temperature. The *R_r_* values of the specimens studied here were 80.9 ± 10.6%, less than the values reported for PET, which were 99.27 ± 0.3 [57]. The decrease in shape recovery for our specimens was most likely driven by the damage incurred by the specimen during room temperature deformation. It is expected that the specimens would have incurred little to no irreversible damage had they been deformed at an elevated temperature; however, as mentioned earlier, PET is not usually applied at an elevated temperature, and we wished our deformation conditions to be examples of a real-world situation.

**Figure 18:**
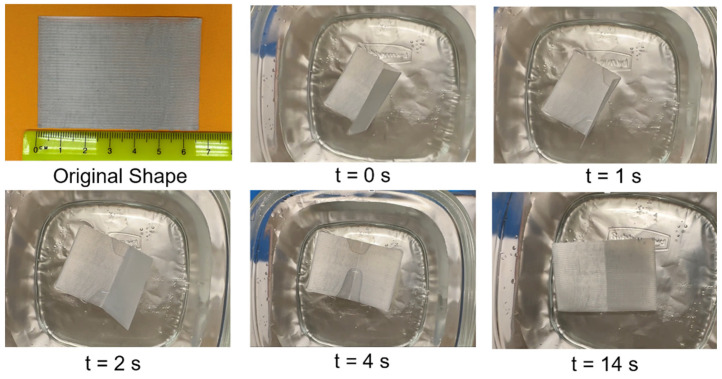
Time lapse of a shape memory experiment involving PET/20% SEBS.

A less quantitative experiment was developed where a sheet of PET was made by way of FFF and then folded in half and placed in water at ~82 °C. As can be seen in Figure 18, the specimen unfolded upon contact with the warm water; the process took a total of 4 s. Though the crease of the fold did not completely disappear, the recovery process was shown to be rapid; the unfolding process occurred immediately upon contact with the warm water. A video of this experiment can be found at the Supplementary Materials: https://youtu.be/R0n4xTYVvWo (accessed date 4 December 2024). 

## 4. Conclusions

The problem of plastic waste is a large societal problem that PET is a small part of. We have demonstrated that discarded drinking bottles could be converted into feedstock for FFF additive manufacturing platforms and that the addition of SEBS imparts an ability to recover from room temperature damage that would potentially otherwise lead to a component made from this material being discarded into the waste stream. This work also demonstrates the benefit of incorporating upcycling and advanced manufacturing methodologies into waste management and reduction efforts. From the experiments performed in this study, the following conclusions can be drawn:

(1) Combining SEBS-g-MA with recycled PET derived from water bottles yields a feedstock that is compatible with FFF additive manufacturing platforms.
(2) The addition of the SEBS content did not yield a large difference in terms of mechanical performance.
(3) Microanalysis of the fracture surfaces of tensile specimens composed of the blends revealed that the SEBS did not adhere well to the PET matrix as SEBS nodules as well as tear-out sites where the nodules once were. This finding was somewhat unexpected, as the grade of SEBS used here possessed a maleic anhydride graft which should have enhanced the bonding between the PET and SEBS; this indicates that the graft was not sufficient to overcome the effects of differing Hildebrand solubility parameters.
(4) Annealing the specimens appeared to promote an improvement in the mixing between the two components, as there was far less evidence of SEBS nodules and tear-outs on the fracture surfaces of annealed specimens.
(5) Further indication that annealing changes the nature of the blend was provided by the chemical differences observed in the FTIR-ATR spectra when comparing annealed and non-annealed (as-printed) specimens.
(6) Additional evidence of the benefits of the annealing process was the more predictable behavior of impact strength, which increased as the SEBS content increased.
(7) The downside of the annealing process was that it led to shrinkage of the specimens as well as potential volatilization of the SEBS, leading to the formation of voids within the material.
(8) The addition of SEBS enabled quantifiable shape memory properties for a blend composed of 20% by mass SEBS.
(9) The PET/20% by wt. SEBS blend did exhibit an ability to recover from room temperature damage, indicating that components made from this material could potentially experience a longer lifespan, leading to a reduction in plastic waste.

## Figures and Tables

**Table 1:** Extrusion parameters.

Parameter	Value
Extruding temperature (°C)	260–265
Rotating speed (rpm)	35
Airpath cooler speed (rpm)	100

**Table 2:** FFF machine parameters.

Parameter	PET and PET-SEBS Blends
Nozzle diameter (mm)	0.5
Nozzle temperature (°C)	265
Bed temperature (°C)	70
Printing speed (mm/min)	1000
Infill (%)	100
Layer height (mm)	0.2
Outline Overlap (%)	99

**Table 3:** Machine parameters used for DMA testing of the materials studied here.

Parameter	Value
Initial temperature (°C)	−80
Final temperature (°C)	180
Heating rate (°C/min)	2
Frequency (Hz)	1

**Table 4:** Critical values gleaned from DMA testing.

DMA Results							
SEBS %	Sample Size (n)	Glassy Onset Temperature (°C)	σ	tan δ Temp. (°C)	σ	tan δ	σ
0% (PET)	4	65.9	2.2	82.5	0.9	1.6	0.2
5%	3	68.1	1.8	81.6	0.3	1.3	0.1
10%	4	67.7	1.1	81.1	0.4	1.3	0.1
20%	4	68.7	2.3	82.5	0.4	1.2	0.1

**Table 5:** Tensile test values.

As Printed					
SEBS (%)	Sample Size (n)	UTS (MPa)	σ	Elongation at Break (%)	σ
PET (0%)	5	51.0	6.6	12.6	4.1
5%	5	40.1	3.9	17.9	13.1
10%	5	39.0	1.2	8.3	0.3
20%	4	35.5	6.2	16.9	10.6
Annealed					
PET (0%)	5	27.4	6.0	3.2	0.7
5%	6	51.6	6.8	6.9	0.6
10%	6	36.7	6.2	5.3	1.2
20%	6	40.5	4.3	6.9	2.0

## Data Availability

The original contributions presented in the study are included in the article/Supplementary Material, further inquiries can be directed to the corresponding author.

## Supplementary Materials

A video corresponding with Figure 18 can be found at the following link: https://youtu.be/R0n4xTYVvWo.
